# Identifying biomarkers for tDCS treatment response in Alzheimer’s disease patients: a machine learning approach using resting-state EEG classification

**DOI:** 10.3389/fnhum.2023.1234168

**Published:** 2023-10-04

**Authors:** Suellen Marinho Andrade, Leandro da Silva-Sauer, Carolina Dias de Carvalho, Elidianne Layanne Medeiros de Araújo, Eloise de Oliveira Lima, Fernanda Maria Lima Fernandes, Karen Lúcia de Araújo Freitas Moreira, Maria Eduarda Camilo, Lisieux Marie Marinho dos Santos Andrade, Daniel Tezoni Borges, Edson Meneses da Silva Filho, Ana Raquel Lindquist, Rodrigo Pegado, Edgard Morya, Seidi Yonamine Yamauti, Nelson Torro Alves, Bernardino Fernández-Calvo, José Maurício Ramos de Souza Neto

**Affiliations:** ^1^Aging and Neuroscience Laboratory, Federal University of Paraíba, João Pessoa, Paraíba, Brazil; ^2^Center for Alternative and Renewable Energies (CEAR), Department of Electrical Engineering, Federal University of Paraíba, João Pessoa, Paraíba, Brazil; ^3^Laboratory of Ergonomics and Health, Department of Physiotherapy, Federal University of Paraíba, João Pessoa, Paraíba, Brazil; ^4^Department of Information Technology, Federal University of Ceará, Crateús, Brazil; ^5^Department of Physiotherapy, Federal University of Rio Grande do Norte, Natal, Rio Grande do Norte, Brazil; ^6^Edmond and Lily Safra International Institute of Neurosciences (IIN-ELS), Macaíba, Rio Grande do Norte, Brazil; ^7^Department of Psychology, Federal University of Paraíba, João Pessoa, Brazil; ^8^Department of Psychology, Faculty of Educational Sciences and Psychology, University of Cordoba, Córdoba, Spain; ^9^Maimonides Biomedical Research Institute of Cordoba (IMIBIC), Córdoba, Spain

**Keywords:** Alzheimer’s disease, electroencephalography, transcranial direct current stimulation, artificial intelligence, machine learning

## Abstract

**Background:**

Transcranial direct current stimulation (tDCS) is a promising treatment for Alzheimer’s Disease (AD). However, identifying objective biomarkers that can predict brain stimulation efficacy, remains a challenge. The primary aim of this investigation is to delineate the cerebral regions implicated in AD, taking into account the existing lacuna in comprehension of these regions. In pursuit of this objective, we have employed a supervised machine learning algorithm to prognosticate the neurophysiological outcomes resultant from the confluence of tDCS therapy plus cognitive intervention within both the cohort of responders and non-responders to antecedent tDCS treatment, stratified on the basis of antecedent cognitive outcomes.

**Methods:**

The data were obtained through an interventional trial. The study recorded high-resolution electroencephalography (EEG) in 70 AD patients and analyzed spectral power density during a 6 min resting period with eyes open focusing on a fixed point. The cognitive response was assessed using the AD Assessment Scale–Cognitive Subscale. The training process was carried out through a Random Forest classifier, and the dataset was partitioned into *K* equally-partitioned subsamples. The model was iterated *k* times using K−1 subsamples as the training bench and the remaining subsample as validation data for testing the model.

**Results:**

A clinical discriminating EEG biomarkers (features) was found. The ML model identified four brain regions that best predict the response to tDCS associated with cognitive intervention in AD patients. These regions included the channels: FC1, F8, CP5, Oz, and F7.

**Conclusion:**

These findings suggest that resting-state EEG features can provide valuable information on the likelihood of cognitive response to tDCS plus cognitive intervention in AD patients. The identified brain regions may serve as potential biomarkers for predicting treatment response and maybe guide a patient-centered strategy.

**Clinical Trial Registration:**

https://classic.clinicaltrials.gov/ct2/show/NCT02772185?term=NCT02772185&draw=2&rank=1, identifier ID: NCT02772185.

## 1. Introduction

Alzheimer’s disease (AD) is a neurodegenerative condition that progressively impairs cognitive abilities, resulting in a loss of personal autonomy and dependence (Sperling et al., 2011; Atri, 2019; Knopman et al., 2021). Transcranial direct current stimulation (tDCS) has emerged as a non-invasive and non-pharmacological alternative for treating cognitive impairment and improving the quality of life in AD patients (Cammisuli et al., 2021). It is suggested that tDCS modulates GABAergic and glutamatergic pathways, as well as mechanisms related to long-term potentiation (LTP) and long-term depression (LTD) associated with plasticity (Pini et al., 2022).

To date, several studies have demonstrated the superiority of active tDCS over sham stimulation in inducing clinical and cognitive changes in AD patients (Saxena and Pal, 2021; Teselink et al., 2021; Andrade et al., 2022). However, results across studies have shown heterogeneity, as noted by Grønli et al. (2022). A systematic review and meta-analysis investigating the effects of non-invasive neurostimulation on healthy older adults and individuals with AD revealed that the optimal timing for tDCS administration and methodological parameters vary based on physiological and pathological aging (Hsu et al., 2015). Furthermore, other researchers emphasize the importance of understanding individual factors that determine responsiveness (Wiethoff et al., 2014; Cruz Gonzalez et al., 2018).

Different functional neuroimaging techniques have been employed to detect alterations in brain activity following tDCS treatment, with Electroencephalography (EEG) playing a crucial role in comprehending the underlying neurophysiological states associated with neuropsychiatric disorders and facilitating identification of biomarkers and diagnostic tools for these conditions (Al-Kaysi et al., 2017). EEG in individuals with AD reveals an increase in widespread delta and theta activity, along with a reduction in posterior alpha and beta activity, which are distinctive features primarily observed during the moderate stages of the disease (Hsiao et al., 2013; Tsolaki et al., 2014). Moreover, studies demonstrate a gradual loss of cortical asymmetry throughout life, with this process being more accelerated in AD (Cabeza, 2002; Roe et al., 2021).

Prior research has also indicated that EEG oscillatory activity exhibits distinguishing patterns in responders to non-invasive brain stimulation compared to non-responders across various neuropsychiatric disorders (Woźniak-Kwaśniewska et al., 2015; He et al., 2020; Metin et al., 2020). Additionally, our recent findings indicate that anodal tDCS combined with cognitive stimulation enhances overall cognitive function and induces changes in EEG brain activity in patients with AD when compared to sham treatment. The alterations in cognitive performance are associated with modifications in EEG measures of brain activity in individuals with AD (Andrade et al., 2022).

Artificial intelligence (AI) has the potential to revolutionize medical practice by enhancing diagnosis, prognosis, and medical care planning as clinical decision support (Esteva et al., 2019; Romero-Brufau et al., 2020). However, implementing AI in healthcare necessitates addressing concerns, risks, and challenges associated with AI technologies (Esmaeilzadeh, 2020; Laï et al., 2020). AI-enabled tools have proven beneficial in the early diagnosis and personalized treatment of AD through analyzing EEG patterns and monitoring disease progression and treatment response (Klöppel et al., 2012). Machine Learning (ML) is a subfield of AI that focuses on algorithms enabling computers to learn from data. ML empowers AI systems to learn and improve automatically, without being explicitly programmed for specific tasks (Esteva et al., 2019). Moreover, AI techniques can effectively integrate multiple features to predict clinically significant outcomes, making them suitable for developing models to accurately determine if an individual patient would benefit from tDCS (Khosla et al., 2019; Shah et al., 2019).

Recent years have witnessed a surge of interest in applying machine learning (ML) to AD detection, owing to its potential in enhancing understanding and diagnosis. Yu et al. (2022) demonstrated ML’s efficacy through the Tensorizing GAN with High-order Pooling (THS-GAN) model, leveraging structural brain image information. Furthering this, Yu et al. (2023) introduced the Multidirectional Perception Generative Adversarial Network (MP-GAN) to visualize AD-related features across patient stages. Lei et al. (2022) presented a deep-learning framework linking brain regions and longitudinal data to predict AD clinical scores. These innovations collectively hold promise for refined diagnostic approaches.

Concurrently, AI-driven efforts have also been directed toward identifying responders and non-responders to interventions, such as tDCS. Paul et al. (2022) ventured into this domain by demonstrating that brain connectivity patterns derived from resting-state functional magnetic resonance imaging can predict the response of persistent auditory verbal hallucinations to tDCS in schizophrenia patients. Similarly, Kayasandik et al. (2022) applied ML analysis to forecast the cognitive effects of repetitive transcranial magnetic stimulation (rTMS) in AD patients via EEG signal analysis. However, the need for further research with larger sample sizes, randomized controlled trials, and longitudinal follow-up is evident, as indicated by the limitations in the study design.

The variability in findings from resting-state EEG studies concerning brain regions implicated in AD emphasizes the necessity to pinpoint specific neural areas affected in individuals with the disease. This determination is pivotal for distinguishing between intervention responders and non-responders. Existing research has uncovered distinct EEG patterns associated with AD. Noteworthy among these are increases in slow oscillations accompanied by reductions in fast oscillations (Andrade et al., 2018; Jafari et al., 2020), as well as excessive prevalence of delta waves and significant decline in posterior alpha rhythms (Franciotti et al., 2020). Conversely, emerging data propose elevated high-frequency oscillations, particularly in beta and gamma ranges, alongside diminished low-frequency oscillations, particularly in frontocentral regions (Huang et al., 2000). Furthermore, irregularities in peak frequency, power, and interrelatedness of alpha, delta, and theta rhythms are noted in relation to disease progression and interventions (Gaubert et al., 2019; Babiloni et al., 2021).

Given the potential of these EEG patterns to unveil unique cerebral functional attributes linked to AD, their investigation assumes paramount importance. Such an approach facilitates the identification of distinctive patterns and potential deviations in brain activity associated with the disease. This, in turn, plays a crucial role in categorizing individuals as intervention responders or non-responders. The inherent heterogeneity in neurodegeneration among AD patients underscores the significance of biomarkers associated with positive responses. These biomarkers can pave the way for precision-targeted treatments, enhancing their effectiveness and efficiency. Consequently, the pursuit of intervention response biomarkers transcends the study of response patterns, offering invaluable tools for tailoring AD treatment and advancing our understanding of disease progression (Ouchani et al., 2021).

Against this backdrop, the primary objective of our investigation is to delineate the cerebral regions implicated in AD, addressing the existing gaps in our comprehension of these regions. To achieve this goal, we have harnessed the power of supervised machine learning algorithms to predict the neurophysiological outcomes resulting from the amalgamation of tDCS therapy and cognitive intervention. Our focus rests on discerning between responders and non-responders to antecedent tDCS treatment, stratified based on cognitive outcomes. At the heart of our approach lies the spectral power analysis of EEG during the quiescent resting state at the inception of the study. In tandem, we emphasize the identification of principal cerebral loci associated with these outcomes. Our hypothesis posits the existence of a subset of discerned features with optimized predictive potential, thereby facilitating the identification of functional biomarkers conducive to prognosticating the cognitive response triggered by the confluence of tDCS therapy and cognitive intervention.

## 2. Methods

### 2.1. Study design

The data set used in this study was collected as part of a randomized, double-blind, placebo-controlled clinical trial designed to compare the efficacy of tDCS and cognitive intervention in patients with AD have been previously published by our research group (Andrade et al., 2018). Participants were randomly assigned to one of four groups: (a) active tDCS plus active cognitive intervention; (b) sham tDCS plus active cognitive intervention; (c) active tDCS plus placebo cognitive intervention; and (d) sham tDCS plus placebo cognitive intervention. In this study, tDCS was applied to six cortical areas affected by AD. These sites are primary centers involved in the manifestation of clinical symptoms of the disease, including the left and right portions of the dorsolateral prefrontal cortex, related to short-term and long-term memory, judgment ability and executive functions; Broca’s area and Wernicke’s area, located in the temporal lobe, responsible for language; and the right and left somatosensory association cortex, in the parietal lobe, related to topographical and spatial orientation and praxis. The choice of these areas follows previously-tested neurostimulation protocols in patients with AD (dos Santos Moraes et al., 2006; Passeri et al., 2022).

The non-pharmacological therapy was administered three times a week for a period of 2 months (24 sessions), and ADAS-Cog assessments were conducted within 7 days of the pre-and post-intervention phases.

By using ML models, we aimed to use the EEG oscillatory activity to predict the response of patients with AD to treatment with tDCS combined with cognitive intervention. Responders were previously defined as patients who exhibited a minimal clinically relevant change (MCRC) on the Alzheimer’s Disease Assessment Scale–Cognitive Subscale (ADAS-Cog) during the post-intervention phase (“ADAS-Cog pre” minus “ADAS-Cog post” ≥ 3.76), as indicated in previous tDCS clinical studies (Andrade et al., 2022). On the other hand, non-responders were defined as patients who did not show an MCRC (“ADAS-Cog pre” minus “ADAS-Cog post” ≤3.76). After this classification of groups based on clinical data, we proceeded to classify the neurophysiological data (EEG) using our ML technique, to confirm the classification of responders or non-responders provided by the clinical data and to analyze which regions would be more implicated for this classification.

### 2.2. Participants

The sample was composed of 70 participants diagnosed with AD according to NINCDS-ADRDA (McKhann et al., 1984), presenting a score of 1 or 2 on the Clinical Dementia Rating (CDR) (Hughes et al., 1982), as well as a score of higher than 12 on the Mini Mental State Examination (MMSE) (Folstein et al., 1975). This study was approved by the institutional ethics committee and conducted in accordance with the principles outlined in the 1964 Declaration of Helsinki. Written informed consent was obtained from all patients or their surrogates before the experiment.

### 2.3. Data collection

EEG was initially conducted in a controlled environment with the patient in a resting state under quiet conditions to obtain the pre-treatment electrophysiological measurements. A BrainVision actiCHamp^®^ device (Brain Products, Munich, Germany) was utilized, featuring 32 channels with active Ag-AgCl electrodes. These electrodes were applied to the scalp using an adjustable cap, following the international 10–20 EEG system, to enable bilateral monitoring of the prefrontal, frontal, temporal, parietal, and occipital regions. The specific channels employed were: Fc1, Fc2, Fc5, Fc6, Fp1, Fp2, F3, F4, F7, F8, FT9, FT10, C3, C4, CP1, CP2, CP5, CP6, T7, T8, P3, P4, P7, P8, O1, and O2. Recording commenced once all electrodes achieved impedance below 10 kΩ, which took approximately 6 min. The recording included alternating rest periods with eyes open while focusing on a fixed point, with each period lasting 1 min. The data were subsequently processed using the Brain Vision Analyzer software program (Brain Products, Munich, Germany), including artifact inspection and removal. Following data segmentation, the Fast Fourier Transform was applied to calculate the power spectra of the delta, theta, alpha, beta, and gamma frequency bands.

### 2.4. Statistical analysis

Taking into account the objectives of the study, a detailed analysis of supervised learning ML algorithms was conducted. This approach is characterized by having prior knowledge of the output variable through learning from past values of the target variable (Bystad et al., 2016). Within this framework, the optimal subset of EEG features was identified using the Random Forest (RF) feature importance tool. This metric represents the relative importance of each variable for the prediction model (Inagawa et al., 2019).

#### 2.4.1. Random Forest method

**Figure 1:**
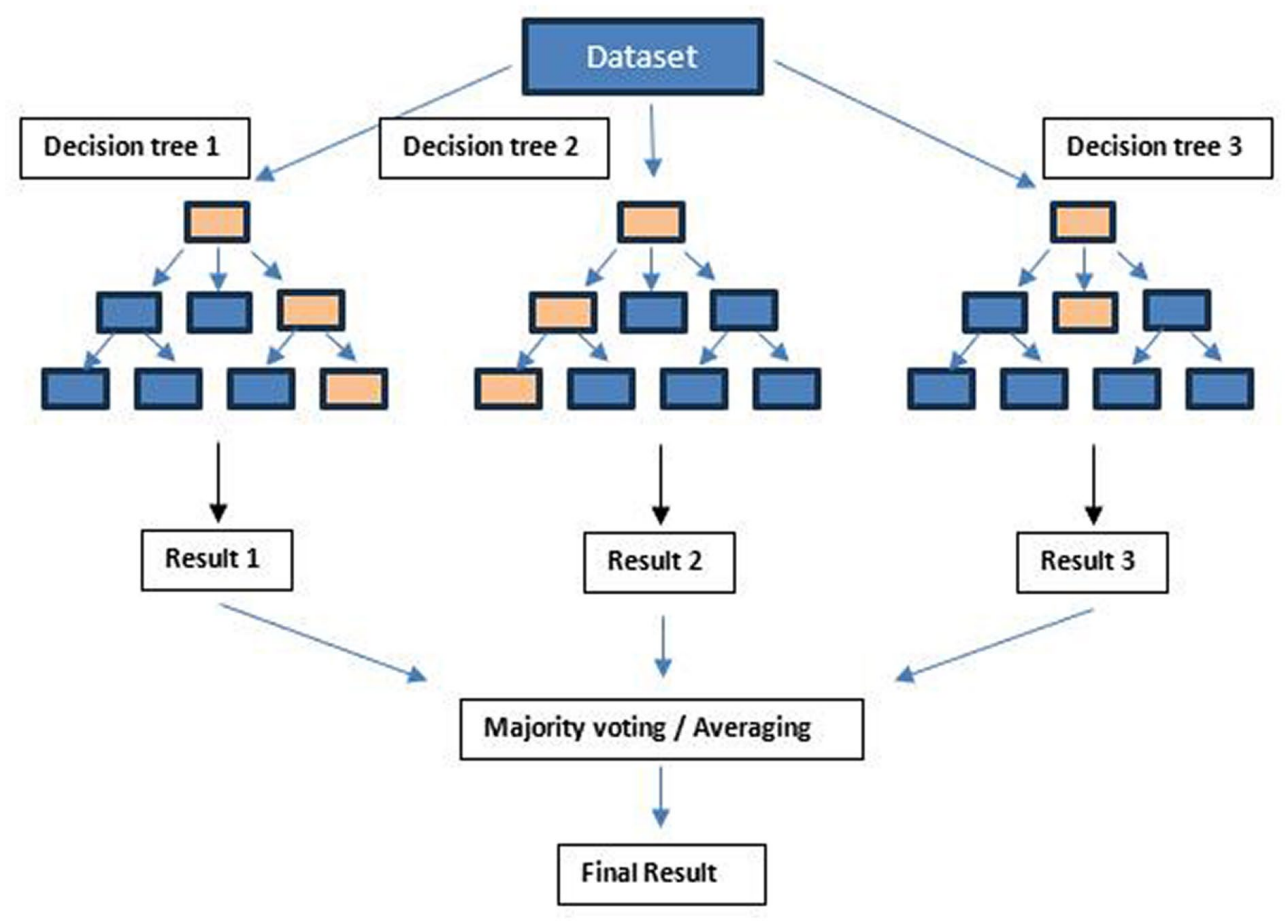
Random Forest working diagram representation.

The Random Forest algorithm by Breiman (2001) is a powerful strategy for classification and regression tasks, employing multiple decision trees to improve accuracy and prevent overfitting. Each tree follows a flowchart structure, using binary feature-based splits to predict the target variable (Figure 1).

**Figure 2:**
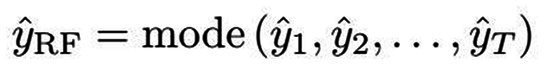
The mathematical representation of Random Forest's final classification prediction.

**Figure 3:**
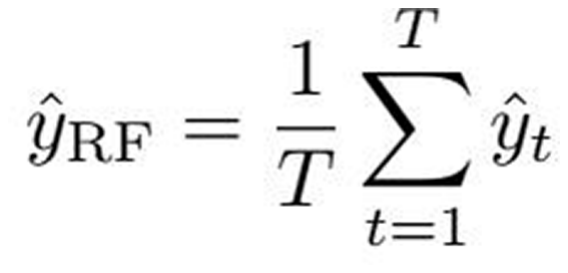
The Random Forest prediction equation.

Through bagging, the algorithm combines these trees by creating subsets of training data, reducing variance. It also fosters diversity by randomly selecting features during tree construction, minimizing correlation. Typically, the square root of total features guides feature selection per split. In classification, tree predictions merge through majority voting; in regression, they average, boosting accuracy and resilience. The mathematical representation of Random Forest’s final classification prediction is illustrated in Figure 2. For classification tasks, the Random Forest prediction arises from the amalgamation of individual decision tree predictions using majority voting, symbolized by “mode” (Liaw and Wiener, 2002). In regression scenarios, the ultimate prediction is expressed by the subsequent equation, with yt representing the tth decision tree’s forecast and T signifying the total forest trees (Liaw and Wiener, 2002; Hastie et al., 2009) (Figure 3).

Meticulous data cleaning, modeling, and structuring were conducted during the preprocessing phase to ensure data quality. Exploratory data analysis techniques, including the utilization of boxplots, were employed to examine data distribution and detect any irregularities. The analysis confirmed uniform data distribution without outliers, further affirming the suitability of the Random Forest (RF) method for our study. This is a robust ML approach that combines decision trees and proved to be an optimal choice for our study due to its capabilities in handling intricate and extensive datasets, alleviating overfitting concerns, and offering reliable estimates of variable importance (Breiman, 2001). By harnessing the inherent strengths of the Random Forest method, we developed an algorithm adept at effectively analyzing complex patterns within EEG frequency bands. This facilitated accurate predictions and facilitated insights into the relationship between these oscillations and respondent characteristics.

RF was employed during the training process, capitalizing on its ensemble of decision trees to construct a more precise and stable prediction model. Thus, two primary strategies were implemented to combat overfitting: randomization of data samples and selection of variable subsets during tree node splitting. A thorough evaluation of all variables was conducted at each iteration to identify the most informative variable, thereby ensuring minimal error in tree splitting (Breiman, 2001). RF facilitated the creation of smaller trees that varied based on the presented EEG data, considering a limited number of variables. The final prediction was obtained through aggregating individual predictions from each tree (Cutler et al., 2012). Moreover, parameter optimization was crucial to ensure the robustness of our prediction model (Andrade et al., 2018). Solely evaluating the model based on training data can lead to overfitting, where the model exhibits high performance during training, but struggles when applied to new data. Thus, we implemented the Random Search technique within the RF algorithm in order to mitigate this issue, which depends on hyperparameters that directly impact the model’s performance. The search space was defined based on the Scikit-Learn library (Pedregosa et al., 2011), aiming to establish an efficient strategy for parameter selection.

Next, the RandomizedSearchCV classifier from the Random Forest, developed by the Scikit-Learn library (Probst et al., 2019), was implemented. This approach involved K-fold cross-validation, where the dataset was divided into *K* = 3 equally partitioned subsamples. The model was trained K−1 times during each iteration using the subsamples as the training set, with the remaining subsample serving as the validation set to evaluate the model’s performance. This process was repeated until each subsample had been utilized as the validation set for the other subsamples.

Splitting a dataset into training and test sets is vital because it allows for evaluating how well a model generalizes to new data, preventing overfitting by assessing performance on unseen examples. This separation also aids in hyperparameter tuning, performance metric calculation, analyzing bias and variance, comparing models fairly, and building trust in machine learning results. Test sets provide a reliable basis for evaluating model performance, enabling better decision-making and accurate assessments of model effectiveness. The parameter values were adjusted based on the specific dataset used. Given the relatively small size of the dataset, it was determined that the obtained results aligned with the expected outcomes.

By incorporating this modified data into the RF algorithm, we obtained the parameters of the RF model, and their corresponding values were as follows: (1) The “Bootstrap” parameter was set to “False,” indicating a sampling without replacement of the training data; (2) The splitting criterion used was “gini,” which measures the data impurity at each tree node; (3) The maximum number of features considered for each split was limited to “sqrt,” corresponding to the square root of the total number of features; (4) A total of “223” decision trees were created in the model; (5) The minimum number of samples required to form a leaf node was set to “4,” while the minimum number of samples to perform a split at an internal node was defined as “2”; and (6) The maximum depth of the tree was specified as “50.” These parameters were optimized using the Random Search technique in conjunction with K-fold cross-validation to ensure the proper performance of the RF model in the study.

To judge the performance of the ML classification algorithms, we ran confusion matrices for the calculation of various performance metrics, such as accuracy, precision, recall, and F1-score. These metrics help assess the classifier’s overall performance, including its ability to correctly classify samples, identify true positives, avoid false positives, and detect false negatives. Along with these measures, we carried out response prediction on all patients calculating: the proportion of responders that were correctly classified (i.e., true positives), the proportion of non-responders that were correctly classified (i.e., true negatives), the proportion of responders that were misclassified as non-responders (i.e., false positive) and the portion of non-responders that were misclassified as responders (i.e., false negative).

Additionally, we employed entropy to select brain regions that most significantly contribute to classification. The algorithm gauges data distribution across predictor variables by considering fluctuations within the target variable. Higher entropy values indicate more data disorder, while lower values signify enhanced data organization. The algorithm assesses information gain for each variable, quantifying the correspondence between predictor and target variables. Essentially, it elucidates the extent to which the activity within specific brain regions explicates variations in the target variable, which, in the context of this study, could pertain to cognitive response or disease progression. The brain region that showcases the most substantial information gain is subsequently chosen as the pivotal variable for the initial node of the decision tree. This selection process hinges upon the brain region’s capacity to effectively elucidate the intricacies of the target variable, ultimately paving the way for the commencement of the decision tree construction centered around the most influential brain region.

## 3. Results

All participants were included in the analysis. The baseline characteristics is provided in Table 1. The difference in ADAS-cog scores between baseline and endpoint was significant (mean group difference 7.12 points; 95% CI 11.03–18.75; *p* < 0.002). In 30 of 70 volunteers, ADAS-Cog decreased with the MCRC and these participants were classified as responders. Non-responders remained on a similar level from baseline or increased their ADAS-cog scores after treatment.

**Table 1:** Baseline characteristics of all participants.

Variables	Type of measurement	
Age (years)	M(SD)	77.8 (± 3.6)
Sex		
Female	(N)	32
Male	(N)	38
Instruments		
DAD scores	M(SD)	85.2 (± 2.8)
ADAS-Cog scores	M(SD)	16.4 (± 2.3)
NPI-Q scores	M(SD)	40.8 (± 4.2)
Disease duration (months)	M(SD)	18.5 (± 3.4)
Medications		
Colinesterase inhibitors	(N)	23
Memantine*	(N)	51

ADAS-Cog, Alzheimer’s disease assessment scale-cognitive subscale; DAD, disability assessment for dementia; NPI-Q, neuropsychiatric inventory questionnaire; ChEIs, cholinesterase inhibitors. Descriptive data is presented as M(SD) = mean ± standard deviations; and *N* = number of participants. *Memantine is an FDA-approved medication used in the treatment of Alzheimer’s disease. It belongs to a class of drugs known as N-methyl-D-aspartate (NMDA) receptor antagonists.

The RF model identified four key brain activity points at baseline associated with two frequency bands as the strongest predictors of cognitive response to tDCS combined with cognitive intervention in AD patients. These findings are presented in Table 2, along with their respective feature importance.

**Table 2:** Brain regions with the highest predictive scores for response to neurostimulation combined with cognitive stimulation in AD patients.

Brain region	Average amplitude (μV)	Feature importance
Frontal	<= 0.959	0.074
Central/parietal	<= 0.995	0.069
Temporal	<= 0.995	0.022
Occipital	<= 1.02	0.051

Average amplitudes refer to the mean of the patterns or oscillations of electrical brain activity at different frequencies (delta, theta, alpha, beta, and gamma) for each brain region. Feature importance corresponds to the frequency of the brain region’s appearance across all trees. The range of feature importance values in a Random Forest can vary from 0 to 1, with higher values indicating greater importance and being considered more influential in making accurate predictions.

**Figure 4:**
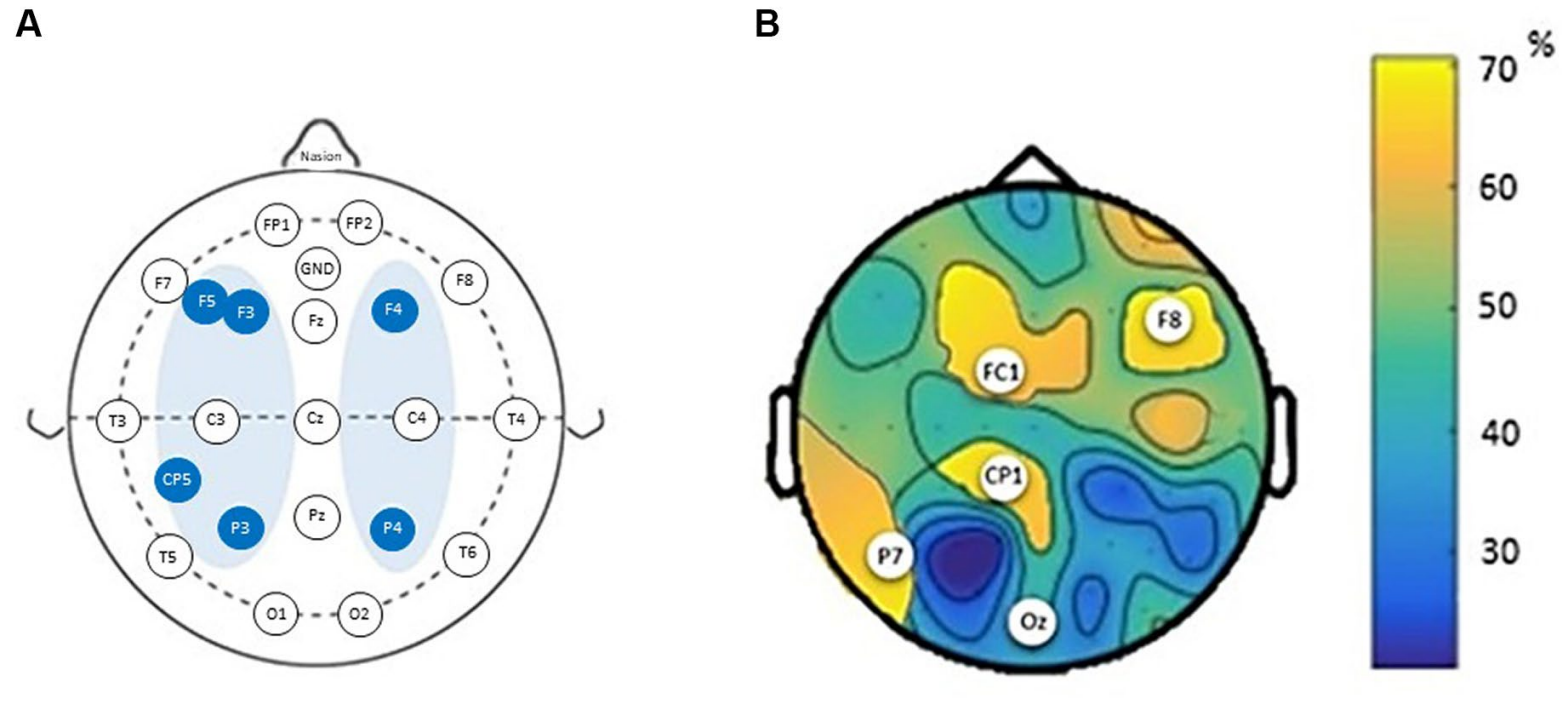
The average classification error for the best channel combinations per brains regions. **(A)** Montage of transcranial direct current stimulation (tDCS) sessions according to the EEG 10/20 System. **(B)** Classification accuracy of individual channels based on cognition labels. White dots indicate the channels with highest accuracy.

To investigate the channels that best predict the treatment response, we then compared the error rates across regions. The classification accuracy of individual channels is shown in Figure 4. The results indicate that the channels located near or inside the brain stimulation area yielded the highest classification accuracy: FC1 (71 ± 4%), F8 (72 ± 8%), CP1 (69 ± 3%), Oz (62 ± 11%), and P7 (64 ± 6%). The average error was lowest for frontal, parietal-occipital area (*p* < 0.01).

## 4. Discussion

In this study the objective was to delineate baseline resting-state EEG features capable of effectively discriminating between intervention responders and non-responders among AD patients, while pinpointing the specific cerebral regions most implicated in this classification. Employing a random forest classifier, we leveraged baseline EEG spectral power to prognosticate the cognitive improvement outcomes subsequent to the intervention. Through comparisons of average classifications encompassing all cerebral regions, the frontal and parietal-occipital channels exhibited superior performance in predicting treatment response. Notably, this study represents the inaugural endeavor, to the best of our knowledge, in successfully applying EEG-based machine learning to predict individual responses to brain stimulation therapy for AD.

Given the complexity of AD, treatment of patients remains challenging (Passeri et al., 2022). Current prediction models of cognitive response in AD patients are typically based on clinical measures and the underlying mechanisms of recovery are still poorly understood. Therefore, implementing quantitative EEG presents a promising biomarker for investigating the neurophysiological impacts of non-invasive brain stimulation techniques. We recently showed that individuals with AD who received active tDCS in combination with cognitive stimulation exhibited enhanced cognitive performance and greater alterations in EEG spectral power and bilateral EEG coherence in comparison to those in the sham group (Andrade et al., 2022). The current study showed that five channels held predictive information of cognitive improvement following a tDCS treatment. The channels identified corresponded with cortical regions stimulated during treatment. Notably, abnormal EEG activity within the frontal and parietal cortex, the region underlying the channel pairs identified, has previously been shown to predict the cognitive response to pharmacological treatment (Passeri et al., 2022). These patterns of EEG abnormalities in AD patients are characterized by a generalized increase in delta and theta activity, a reduction in posterior alpha and beta activity in the frontal and temporo-parietal areas, with less complex brain activity correlated with severity of dementia (dos Santos Moraes et al., 2006; Babiloni et al., 2013). Moreover, we observed that the accurate EEG outcome classifier confirms frontal and parietal–temporal areas as neuroanatomical marker for brain stimulation. This is a finding of particular interest, as tDCS of the dorsolateral prefrontal cortex, inferior parietal region and temporal lobe has also been used in the experimental treatment of Alzheimer’s disease (Van der Hiele et al., 2007; Bystad et al., 2016; Roncero et al., 2017; Inagawa et al., 2019; de Almeida Rodrigues et al., 2020).

Our results further showed that EEG occipital area were predictive of treatment response. This finding may be an indication of the presence of complex and diverse pathway underling AD neurobiology. Interestingly, the reduction in the alpha band and reduced beta power in the parietal and occipital regions has previously been shown to differentiate normal aging, mild cognitive impairment, and AD (Jeong, 2004; Rossini et al., 2006). According to Plaza-Rosales et al. (2023), the core network for navigation is constituted by the hippocampus, parietal, prefrontal, and occipital regions. This network impairment affects several frontal lobe areas and occipitofrontal desynchronization in subjects with AD is part of this compensatory mechanism rather than just a poor visual–spatial ability during cognitive tasks. Further research is necessary to confirm this putative role of occipital area and the clinical utility of this target in tDCS treatment of AD.

Our study provides valuable insights into personalized treatments for AD by conducting a secondary analysis that goes beyond the overall effectiveness of tDCS. We employed a ML approach to focus on patient stratification and predict cognitive response. The resting-state baseline EEG of AD patients proved to be a reliable predictor of cognitive changes, as measured by the ADAS-cog instrument, following treatment with tDCS combined with cognitive intervention. We specifically identified specific cortical areas that were highly predictive in patients who responded to the intervention. Targeting these regions in the intervention could potentially reduce response variability and enhance the effectiveness of these techniques in clinical practice.

According to your results, the brain regions most involved in predicting responders to intervention in AD patients were represented by the channels FC1, F8, CP5, Oz, and F7. FC1 and F8 are situated in the frontal cortex, which plays a pivotal role in cognitive functions such as memory, attention, and executive processes. CP5 is associated with the parietal cortex, involved in spatial processing and attention. Oz corresponds to the occipital cortex, responsible for visual processing, while F7 resides in the frontal-temporal junction, implicated in various cognitive tasks (Huang et al., 2000). Yang et al. (2022) hint at these channels’ relevance in capturing distinct connectivity patterns, which could be perturbed in AD-related disruptions. This aligns with the notion that these channels might offer insights into the structural and functional intricacies characterizing AD. Also, these specific brain regions align with the documented alterations in AD. Frontal regions (FC1 and F8) are affected due to the disruption of communication between various regions, as seen in AD’s cognitive deficits. The parietal cortex (CP5) is implicated in spatial processing deficits, and the occipital cortex (Oz) experiences disruptions in visual processing. The frontal-temporal junction (F7) is relevant to memory and other cognitive functions compromised in AD (Plaza-Rosales et al., 2023). In essence, the convergence of EEG findings, the understanding of AD pathology, and the insight into structural and functional dynamics within specific brain regions provide a comprehensive framework for the selection of FC1, F8, CP5, Oz, and F7 channels. These regions, inherently linked to the cognitive impairments and neural disruptions in AD, emerge as pivotal predictors for assessing tDCS intervention response in AD patients.

The observed amplification of power in brain regions linked to FC1, F8, CP5, Oz, and F7 channels among AD individuals displaying notable treatment responses (more than 4 points gain in ADAS-Cog scores) adds a layer of complexity to treatment efficacy in the context of AD. These regions, essential for cognitive processes encompassing memory, attention, visual processing, and executive functions, are intertwined with several factors intrinsic to AD’s pathophysiology.

The augmented power within regions affiliated with the Default Mode Network (Chen et al., 2023) potentially underscores preserved intrinsic connectivity and engagement in introspective cognitive tasks, suggesting that these regions could be crucial for treatment-induced cognitive enhancements. Enhanced functional integrity within these regions among responders might contribute to their favorable treatment response. Moreover, individuals with substantial cognitive reserve could leverage these regions’ intact neural resources (Raichle et al., 2001), potentially explaining the connection between heightened power and positive treatment outcomes. Additionally, the variance in disease severity might underpin these observations, as responders might belong to a subgroup with milder neurodegeneration, rendering these regions more responsive to treatment-induced improvements. The involvement of plasticity mechanisms (Stern, 2002) is also plausible, as the heightened power might signify the potential for reorganization and adaptation, aligning with the brain’s inherent ability to remodel its functional networks. Altogether, the increased power in these regions among treatment responders highlights their relevance, demanding further investigation to comprehend the intricate interplay between network dynamics, cognitive reserve, disease severity, and plasticity mechanisms within these specific neural substrates.

Specifically, the identified brain regions in this study exhibit a noteworthy relationship with the Default Mode Network (DMN) (Chen et al., 2023). The DMN is recognized as a crucial neural network that becomes compromised early in the course of AD. The posterior brain areas, including the precuneus, which is part of the DMN, have been reported to display hyperexcitability in AD patients (Merzenich et al., 2014). This hyperexcitability reflects synaptic dysfunction, contributing to disruptions in long-range connectivity (Casula et al., 2023). Therefore, the correspondence between our study’s findings and the existing knowledge of hyperexcitability in the DMN-associated posterior regions adds a layer of significance to our results. It suggests that the aberrant activity observed in FC1, F8, CP5, Oz, and F7 could be related to the malfunctioning of the DMN, which might be indicative of underlying AD pathology and offer potential insights into the mechanisms behind the observed treatment response in the context of tDCS interventions combined with cognitive intervention.

The inquiry into the nature of the identified biomarkers prompts an exploration of their attributes and relevance in delineating treatment responders from non-responders. Yang et al. (2022) investigation into person-identifying EEG “base signals” underscores their temporal stability, contextual independence, and consistent distribution across channels. Particularly pronounced within monozygotic twins, these signals suggest a structural basis rather than functional manifestations, extending beyond specific task contexts. Concurrently, Chen et al. (2023) study of rTMS treatment efficacy in Alzheimer’s patients highlights the left angular gyrus as a target for cognitive enhancement. Cognitive improvements correlate with DMN connectivity patterns, indicating a potential interplay between structural and functional disparities—a composite nature intrinsic to the biomarkers. Synthesizing these insights, it is conceivable that the biomarkers identified in rTMS interventions (Chen et al., 2023) encapsulate a blend of structural and functional distinctions between responders and non-responders. These conjectures align with the person-identifying EEG base signals’ potential portrayal of unique connectivity patterns within cerebral networks, delineated by Yang et al. (2022). The reciprocal influence of structural traits and functional dynamics, as evidenced by DMN subsystem connectivity during rTMS interventions, emerges as an influencer of cognitive responses. The identified biomarkers, reflecting both structural and functional aspects of treatment response, could potentially extend their influence to tDCS studies due to the shared underlying dynamics of brain modulation. While the direct nature of tDCS introduces unique variables, the interplay between structure and function highlighted by these biomarkers remains relevant.

However, it is important to acknowledge the limitations of our study. Firstly, it is worth noting that the eligibility criteria for participants in our randomized clinical trial may differ from the patient profiles encountered in routine clinical practice, such as frontotemporal dementia, mild cognitive impairment, and severe AD. To measure the cofounding effects of inter-individual differences, the development of tDCS protocols considering the EEG characteristics would be useful to the future implementation of these ML models in clinical practice. Secondly, it is essential to highlight that our analysis has predominantly centered on EEG data and its associations with cognitive responses among patients with AD. However, it is of utmost significance to acknowledge that pertinent clinical variables, such as age, sex, and baseline cognitive status, were not encompassed within our analysis. To obtain a more holistic grasp of the multifaceted determinants influencing cognitive outcomes in AD, forthcoming investigations should seek to integrate these pivotal variables. Moreover, broadening the scope of our study to encompass larger and more diverse cohorts would undoubtedly bolster the external validity of our findings. Thirdly, while our study maintains an exploratory and neurophysiological focus, it’s important to note that considering all four participant groups could provide deeper insights into the interactions between tDCS treatment, cognitive intervention, and practice effects on ADAS-Cog scores. By doing so, we could better elucidate the potential interplay between tDCS treatment, cognitive intervention, and practice/learning effects, ultimately leading to a more comprehensive understanding of the observed changes in ADAS-Cog scores. However, our analysis is concentrated on tDCS treatment responders, reflecting the study’s exploratory nature and utilization of machine learning techniques.

Overall, our findings underscore the potential of personalized treatments in AD and pave the way for future research to validate and expand upon our results in larger and more diverse cohorts. Indications between augmented power in brain regions found in this study and positive treatment responses in AD hold profound clinical and research implications. These findings underscore the potential of utilizing these regions as biomarkers for treatment responsiveness, allowing for more personalized therapeutic interventions tailored to individual neural profiles. This could enhance treatment efficacy and optimize cognitive improvements in AD patients. From a research perspective, these observations stimulate further investigations into the underlying mechanisms that render these regions more receptive to treatment-induced enhancements.

## 5. Conclusion

Our results support that resting-state EEG dataset could be a viable approach for extracting predictive features of tDCS treatment efficacy in patients with AD. Furthermore, specific cortical regions that are most predictive of the cognitive response to the interventions have been identified. The achieved results hold clinical importance due to the ability to identify cortical regions that would serve as optimal targets for non-invasive brain stimulation in AD treatment. Considering that routine scalp EEG is relatively inexpensive (compared to intracranial EEG and magnetic resonance image) and feasible to acquire in ambulatory settings, our findings can form the basis for novel and cost-effective ways to assist the AD patients, utilizing prognostic biomarkers can guide and optimize the therapeutic application of cognitive treatment.

## Data availability statement

Publicly available datasets were analyzed in this study. This data can be found at: https://www.clinicaltrials.gov/ct2/show/NCT02772185?term=NCT02772185&draw=2&rank=1.

## Ethics statement

The studies involving humans were approved by Ethics committee of the Federal University of Paraíba (CAAE: 44388015.7.0000.5188). The studies were conducted in accordance with the local legislation and institutional requirements. The participants provided their written informed consent to participate in this study.

## Author contributions

SA, LS-S, FF, KM, NA, BF-C and JS are the investigators responsible for project design and protocol writing. SA, CC, EA, EL, FF, MC and JS contributed to the study background, general design, study variable definition, and statistical analysis planning. SA, LS-S, KM, LA, DB, ES, AL, RP, EM, SY, NA, BF-C, and JS contributed to the preparation of the project. All authors contributed to the article and approved the submitted version.
